# Physical fitness profile of military lifeguards

**DOI:** 10.47626/1679-4435-2023-1123

**Published:** 2024-09-24

**Authors:** Francine de Oliveira, Victor Gonçalves Corrêa Neto, Ricardo Castro Ferreira de Mello, Humberto Miranda

**Affiliations:** 1 Laboratório de Desempenho, Treinamento e Exercício Físico, Rio de Janeiro, RJ, Brazil; 2 Programa de Pós-Graduação em Educação Física, Escola de Educação Física e Desportos, Universidade Federal do Rio de Janeiro, Rio de Janeiro, RJ, Brazil; 3 Centro Universitário Gama e Souza, Rio de Janeiro, RJ, Brazil; 4 Universidade Estácio de Sá, Rio de Janeiro, RJ, Brazil; 5 SALUS – Laboratório Integrado de Pesquisas em Exercício, Biomedicina e Saúde Coletiva, Rio de Janeiro, RJ, Brazil

**Keywords:** physical functional performance, exercise, military personnel, occupational fitness, tactical personnel, desempenho físico funcional, exercício físico, militares, saúde do trabalhador, profissionais de segurança pública

## Abstract

**Introduction:**

Throughout a lifeguard’s career, regardless of age, the physical demands of performing a water rescue are likely to remain constant.

**Objectives:**

To establish a physical fitness profile by age group of a sample composed of professional beach lifeguards who regularly engage in physical activity.

**Methods:**

One hundred and seventy-two lifeguards were initially recruited for this study. Data from 99 male lifeguards were provided for analysis. Anthropometric measurements, lower limb strength, and handgrip measurements were taken at the initial visit. During a second visit, running tests were performed on sand. All testing procedures followed the same predefined sequence for all individuals.

**Results:**

One-way analysis of variance with post hoc Bonferroni or Kruskal-Wallis adjusted analysis was performed to assess differences between age groups depending on the exact variable. The significance level was set at p ≤ 0.05. There were statistically significant differences in all power and velocity performance parameters as well as body fat percentage with better results found in the younger age groups (20-29;30-39) compared to an older age group (40-49). However, there were no statistically significant differences between the groups for handgrip strength.

**Conclusions:**

The results of the present study indicate significant differences in physical fitness between different age groups. The authors suggest that the squat jump, countermovement jump, 20-meter linear sprint test, zigzag change-of-direction test, and running anaerobic sprint test assessment should be used as screening tools for fitness.

## Abstract (pt)

**Introdução:**

Ao longo da vida profissional de um guarda-vidas, independentemente da idade, as exigências físicas impostas durante um salvamento provavelmente se manterão constantes.

**Objetivos:**

Estabelecer um perfil de aptidão física de uma amostra composta por guarda-vidas profissionais praticantes de atividade física regular.

**Métodos:**

Cento e setenta e dois salva-vidas foram inicialmente recrutados para este estudo. Dados de 99 indivíduos foram utilizados para análise. Medidas antropométricas foram obtidas durante a primeira visita, assim como a análise da potência de membros inferiores e da força de preensão manual. Testes de velocidade e mudança de direção foram realizados em uma segunda visita. A testagem seguiu a mesma ordem predefinida para todos os indivíduos.

**Resultados:**

Uma análise de variância com *post-hoc* de Bonferroni ou o teste de Kruskal-Wallis com comparações por pares ajustada foi utilizado para comparação entre grupos, a depender da variável. O nível de significância adotado foi de ≤ 0,05. Foram observadas diferenças estatisticamente significativas entre as faixas etárias para todos os testes de potência e velocidade, bem como no percentual de gordura corporal, com melhores resultados encontrados nas faixas etárias mais jovens (20-29 e 30-39 anos quando comparados ao grupo 40-49). No entanto, não houve diferença estatisticamente significativa entre os grupos em relação à força de preensão manual.

**Conclusões:**

Os resultados indicam diferenças estatisticamente significativas na aptidão física de guarda-vidas entre as diferentes faixas etárias. É sugerido que o salto vertical, sem e com contramovimento, o teste de 20 metros, o teste de corrida anaeróbica e o teste em zigue-zague sejam implementados como ferramenta para triagem da aptidão física.

## INTRODUCTION

Because of the nature of their duties, lifeguards may be exposed to periods of sedentary behavior combined with sudden bursts of high-intensity physical demands that require a high level of physical conditioning to meet the demands of water rescue, for example.^1,2^ According to the World Health Organization (WHO), drowning is one of the leading causes of death that can be prevented by early intervention of a lifeguard.^3^

The physical readiness of lifeguards is an important part of the success of the rescue. According to previous research, oxygen uptake, blood lactate, and heart rate increase to near maximum levels during a water rescue, highlighting the importance of strength and aerobic capacity for lifeguards.^2^ Throughout their working lives, regardless of age, the physical demands of performing a water rescue are likely to remain constant. Under this premise, maintaining a good to optimal level of physical fitness of lifeguards should be considered critical.

Body composition is widely used as a measure of health and fitness.^4^ In addition, various fitness components such as strength and agility have been associated with performance in military populations such as firefighters^5^ and police cadets.^4^ While data on the physical fitness levels of police officers and other military personnel are emerging in the scientific literature, there is a lack of similar profiling for the physical fitness of lifeguards. Previous research in other professions has shown a relationship between functional performance and job duties.^4,6^ Thus, it is worthwhile to draw a profile of lifeguards to pave the way for future research that can establish normative values for this specific population and identify characteristics that contribute to their physical fitness and success in marine rescue.

Previous studies have raised the importance of training lifeguards in more specific ways based on good physical and technical skills to enable faster and more effective interventions in potential drowning situations.^7^ However, the lack of information on the physical profile of professional beach lifeguards hinders the development of appropriate test batteries and training regimens, which are critical for identifying suitable candidates, improving performance during rescues, and establishing guidelines for returning to active duty. Therefore, the purpose of this study was to draw a physical fitness profile by age group for a sample composed of professional military beach lifeguards.

## METHODS

One hundred and seventy-two military lifeguards from the city of Rio de Janeiro, state of Rio de Janeiro, Brazil, were initially recruited for this research. Seventy-three individuals did not complete all the proposed assessments because of time constraints or job demands. Six individuals were excluded because of the use of anabolic substances, fifteen were excluded because they could not complete all test procedures for reasons unrelated to the study, and 44 individuals were excluded because they performed regular physical activity outside the battalion. Data from 99 male lifeguards were provided for analysis. All testing procedures were conducted during summer. Familiarization with all testing procedures was provided verbally one week prior to the beginning of data collection. Anthropometric measurements were taken upon initial arrival, as well as lower limb strength and handgrip measurements. Running tests were performed on sand during a second visit. All testing procedures followed the same predefined sequence for all individuals.

Prior to data collection, approval was obtained from the University Review Board (number 22569119.0.0000.5257). Sample size was calculated considering an error of 0.05, a presumed prevalence of 0.25, and a 95% CI, resulting in a total of 97 individuals.

Inclusion criteria consisted of (a) being a military lifeguard on active duty, (b) abstaining from alcohol, caffeine, and any anti-inflammatory drugs 72 hours prior to testing and throughout the experiment design, and (c) not performing any physical training other than that the one traditionally proposed by the battalion. Exclusion criteria consisted of (a) any type of musculoskeletal injury during the last month prior to data collection, (b) use of anabolic steroids, and (c) a previous history of cardiovascular complications. Participants were categorized into three distinct age groups: 20–29 (n = 34), 30–39 (n = 49), and 40–49 (n = 16). Sample characteristics are displayed in Table 1.

**Table 1:** Mean and standard deviation for age, body mass, height, military service time, BMI, and BF

Age group	Age (years)	Body mass (kg)	Height (m)	Military service time (years)	BMI (Kg/m^2^)	BF (%)
20–29	26.32 ± 2.23	80.51 ± 11.10	1.76 ± 0.07	6.00 ± 2.21	25.71 ± 3.08	13.61 ± 5.56
30–39	34.04 ± 2.64	81.64 ± 10.53	1.76 ± 0.06	9.90 ± 4.43	26.08 ± 3.08	15.01 ± 4.80
40–49	44.06 ± 3.69	86.36 ± 10.99	1.76 ± 0.05	19.81 ± 5.44	27.55 ± 3.27	21.69 ± 13.41

BF = body fat; BMI = body mass index.

### ANTHROPOMETRY

Height was measured using a stadiometer attached to a scale with a maximum height of 200 cm. Participants positioned themselves with their body weight evenly distributed in a hip-width stance at rest, forming a perpendicular angle of 90º with the vertical shaft of the stadiometer.^8^ Weight was measured by using a mechanical scale (Filizola®, São Paulo, SP, Brazil) with a maximum capacity of 150 kg and a precision of 100 g. Body fat (BF) percentage was calculated by using a scientific adipometer (CESCORF®, Porto Alegre, RS, Brazil) with a precision of 0.01 mm, following Pollock’s three-skinfold protocol. Each skinfold was measured twice, and the mean value was used for analysis. Body mass index (BMI) was also calculated. To reduce the margin of error in testing, an experienced evaluator conducted all measurements.

BF percentage was calculated through the following formula^8^:

body density for males=1.1093800−0.0008267(∑3M)+0.0000016(∑3M)−0.0002574(age y)%BF=([4.95/Body Density]−4.5)×100

### SQUAT JUMP (SJ) AND COUNTERMOVEMENT JUMP (CMJ)

Warm-up consisted of lower limb dynamic stretching, ten calf raises, five SJs, and five CMJs.^9,10^ When performing SJs, individuals would start in a half isometric (6 seconds) squat position (90º knee position); when performing CMJs, individuals began in a standing, neutral position. For both tests, individuals would maintain their arms akimbo to avoid contribution of the upper limbs. Five trials were performed for each test, with a 3-second rest interval between trials. The highest jump (in centimeters) was used for statistical analysis. Tests were conducted on a force platform (Force Plate SV – CEFISE® Nova Odessa, SP, Brazil).

### HANDGRIP

Warm-up consisted of ten wrist flexion as well as 10 wrist flexion using a bat, ten light grips and one submaximal grasp on a Jamar handgrip dynamometer (Jamar® Dynamometers, Fabrication Enterprises, NY, USA) used in the second handle position.^11^ Subjects remained seated with their shoulders adducted, and neutrally rotated, with elbows flexed at 90º. Individuals performed three trials with a 15-second rest interval between them holding their maximal voluntary contraction for 5 seconds. The highest value of their dominant limb was used for statistical analysis.

### 20-METER LINEAR SPRINT TEST, ZIGZAG CHANGE-OF-DIRECTION (COD) TEST, AND RUNNING ANAEROBIC SPRINT TEST (RAST)

Warm-up consisted of 5 minutes of submaximal running at a self-selected pace, with two short-duration sprints at the end of the 3rd and 5th minute.^12^ Submaximal attempts were conducted to minimize the learning effect. Performance on the linear sprint test was evaluated by using three photocells (CEFISE®) positioned at 0 and 20 meters. The starting point was positioned 30 meters behind the starting line. Two attempts were conducted with a 5-minute rest interval between them, and the fastest trial was recorded for statistical analysis.^13^ Zigzag COD test consisted of four 5-meters sections (totaling a 20-meter linear sprint). Sections were marked with cones set at 100º angles. Two attempts were conducted with a 5-minute rest interval between them. Individuals positioned themselves 30 meters behind the first pair of photocells (CEFISE®). They were asked to complete the test as quickly as possible until crossing the second pair of photocells. The fastest time was used for statistical analysis.^14^ The RAST evaluation consists of six 35-meter sprints performed as fast as possible with a 10-second rest interval between each sprint.^15^ A pair of photocells (CEFISE®) recorded total test time in seconds and hundredths of seconds. Verbal encouragement was given in a standardized manner throughout all tests.

### STATISTICAL ANALYSES

Statistical analyses were conducted by using SPSS v. 20.0 software (IBM Corp., IL, USA). Data distribution was assessed through the Shapiro-Wilk test, kurtosis and skewness analyses, and graphical observation. When the normality of the data was rejected, a Kruskal-Wallis adjusted analysis was conducted to assess differences between age groups. When data normality was not rejected, a one-way analysis of variance (ANOVA) with post hoc Bonferroni was conducted by observing the homogeneity of variances. The level of significance was set at p = 0.05. Data is reported as mean and standard deviation (SD) for parametric analyses and as median and interquartile range (IQR) for nonparametric analyses.^16^ Correlations between RAST, CMJ, and SJ were also conducted. Values > 0.70 are be regarded as “strong” correlations, values between 0.50 and 0.70 as “good” correlations, between 0.30 and 0.50 as “fair” or “moderate” correlations, and any value < 0.30 as poor correlations.^17^ A one-sample *t* test will be conducted when needed to compare the present sample with available data.

## RESULTS

There were no statistically significant differences in BMI between the groups. However, a statistically significant difference was found for BF percentage between the age groups “20–29” and “40–49” as well as between the groups “30–39” and “40–49”.

Data regarding vertical jump performance and differences between the distinct age groups are displayed in Table 2. Handgrip performances and differences between groups are shown in Table 3. Finally, performance on velocity-based tests and their respective differences between age groups are displayed in Table 4.

**Table 2:** Performance on SJ and CMJ divided by age group and presented as mean and standard deviation

Age group	SJ (cm)	CMJ (cm)
20–29 (n = 34)	24.52 (± 2.90)	27.19 (±3.22)
30–39 (n = 49)	24.50 (± 3.33)	27.45 (± 4.32)
40–49 (n = 16)	20.68 (± 3.53)*	23.49 (± 4.47)*

CMJ = countermovement; SJ = squat jump.

* Significant differences between age groups.

**Table 3:** Performance on handgrip divided by age group and presented as mean and standard deviation

Age group	Handgrip (kgF)
20–29 (n = 34)	58.18 (± 7.72)
30–39 (n = 49)	60.20 (± 771)
40–49 (n = 16)	59.88 (± 8.64)

**Table 4:** Performance on the velocity tests divided by age group and presented as mean and standard deviation

Age group	Zigzag COD (sec)	20-meter sprint (sec)	RAST (sec)
20–29 (n = 34)	5.66 (± 0.35)	3.48 (± 0.21)	39.32 (± 2.66)
30–39 (n = 49)	5.58 (± 0.32)	3.48 (± 0.21)	40.57 (± 3.45)*
40–49 (n = 16)	5.92 (± 0.38)*	3.67 (± 0.31)*	43.84 (± 5.15)*

COD = Change-of-direction; RAST = Running anaerobic sprint test.

* Significant differences between age group.

Nonparametric Spearman correlations revealed a negative correlation between RAST and CMJ as well as between RAST and SJ (Table 5).

**Table 5:** Correlations between RAST, CMJ, and SJ performance

	RAST			
	R	Classification	p-value	r^2^
CMJ	−0.473	moderate	0.000	0.22
SJ	−0.433	moderate	0.000	0.18

CMJ = Countermovement jump; RAST = Running anaerobic sprint test; SJ = squat jump.

## DISCUSSION

The purpose of this study was to draw the physical fitness profile of professional beach lifeguards by age group. Statistically significant differences were found in all power and velocity performance parameters as well as BF percentage, with better results found in the younger age categories (i.e., 20−29 and 30−39) when compared to the older age group (i.e., 40−49). However, no statistically significant differences were found between the groups regarding handgrip strength. A considerable change in BF over the years is closely associated with changes in body weight across middle-aged individuals in general population.^18^ However, this trend was not observed in our data. While BF percentage may generally increase with age, this increase may not be significant enough to markedly change body weight in a sample that probably has a high rate of energy expenditure, considering its daily demands.^7,19^

Considering that there may be an increase in water rescues during summer, the ability to perform maximal sprints of short duration may be essential. Thus, performance on the RAST may serve as an important screening tool for readiness. Apparently, the age-associated decline in men’s anaerobic performance is steeper than in aerobic power,^20^ which was partially supported by the results of this analysis. The association between RAST and vertical jump was observed in a sample of young futsal players.^15^ This may be the reason why lower limb performance also declined with age in the present sample. A possible association between RAST and vertical jump performance in a sample of professional beach lifeguards may permit that CMJ and SJ tests can be used as alternative testing tools. Power testing may also be an option for assessing one’s ability to accelerate, as covering a distance in a short time is highly dependent on power performance.^21^ This is supported by the results of the present study, which showed that the older age group (i.e., 40−49 years) had the worst performance on the 20-meter sprint test.

Physically demanding jobs also require an optimal level of COD ability, which relies on several components, such as technique, sprinting speed, strength, and power.^22^ Zigzag COD, 20-meter sprint test, and RAST were performed on the beach in order to reflect the specificity of their daily work environment. This is not a limitation because, according to previous studies, activities that involve zigzag COD ability performed on sand require different motor and biomechanical strategies compared to flat surfaces.^23^ In addition, sprint activities can be performed at maximum speed with greater energy expenditure and less joint impact on sand surfaces. In addition, COD-type activities can achieve higher values during the deceleration phase.

As expected, the older age group presented poorer results on the zigzag COD test, probably due to the decline in strength and power associated with the aging process, which begins subtly in middle age.^24^ However, this decline is not abrupt,^24^ as indicated by the mean values presented in this sample. This natural decline in performance due to the aging does not necessarily represent an inability to perform their job duties, as lifeguards are likely to have levels of physical readiness than the overall population. This is hypothesized because lifeguards are required to pass specific physical fitness tests as a prerequisite for employment.

When comparing the results of the present study to other military samples, a similar trend is observed in variables such as vertical jump performance. We conducted a one-sample *t* test to compare our data with the results of Perroni et al.^6^ Young military firefighters were found to perform significantly better than military lifeguards (p < 0.001). This could be due to the older range of lifeguards in our sample (20−29 years), whereas Perroni et al.^6^ included younger individuals (20−22 years). However, when comparing older age groups, there is no statistically significant difference between the samples (p = 0.067). This suggests a trend of declining performance that exists regardless of the specificity of job.

When evaluating large groups quantitatively, instruments or tests that provide quick, easy measurements are extremely useful. For example, the use of BMI to classify obesity in epidemiologic studies is often preferred to methods that assess body composition.^25,26^ Although all groups in the present sample had BMI values above the healthy cutoff and were classified as overweight, these values differ from those reported by Perroni et al.^6^ A one-sample *t* test showed statistically significant differences between all categories. Please note BMI is strongly influenced by high levels of fat-free mass. However, data from Perroni et al.^6^ did not include information on fat-free mass or BF percentage, thereby limiting comparisons.

The use of the handgrip strength as a marker of muscle strength may be a feasible option. Handgrip strength was associated with task performance in a sample of police recruits^27^: those with lower grip strength scores were more likely to fail specific occupational task assessments. Although a different sample was studied, both populations present similar occupational demands, as both are required to perform dynamic tasks, such as pushing, grabbing, lifting, and carrying. In the present study, there were no statistically significant differences in handgrip strength between the groups. Even without statistically significant differences in handgrip performance, a complete characterization of one’s strength profile requires testing of both the lower and upper limbs.^28^ Furthermore, isometric handgrip strength is only one type of strength required in their jobs. The present study was not limited to handgrip strength and also assessed power performance of lifeguards, which is an important manifestation of dynamic strength associated with performance.^29^

The current study has some limitations, such as sample differences between age groups. This may be due in part to the fact that older lifeguards may be assigned to duties that do not directly involve water rescue. In addition, we could not control for the specific physical demands of each lifeguard, as these rely on mischance. Another limitation relates to the collection of data on the use of anabolic agents; no urine or blood samples were collected, relying instead on anamnesis for collecting data on the use of performance-enhancing substances. Furthermore, because of the possibility that one’s performance may oscillate throughout the seasons, potential biases were controlled by collecting data in a single season of the year.

## CONCLUSIONS

In conclusion, our findings indicate significant differences in physical fitness, except for handgrip performance, across different age groups of beach lifeguards. Therefore, we propose that SJ, CMJ, 20-meter linear sprint test, zigzag COD Test, and RAST assessment should be implemented as a screening tool for readiness. Furthermore, in view of the natural aging process, we suggest that complementary, individualized training programs should be implemented in order to mitigate natural declines in physical aptitude.
